# Supplementary material for: Modeling glioblastoma heterogeneity as a dynamic network of cell states
Source: Mol Syst Biol. 2021 Sep 16;17(9):e10105. doi: 10.15252/msb.202010105 (PMC8444284; doi:10.15252/msb.202010105)
Supplement: Supplementary file 5 — Source Data for Figure 3 [file MSB-17-e10105-s001.zip › Figure3A_sourcedata/GSEA_3065/nosigdb_state1.GseaPreranked.1622718409535/DOROSZKO_U3180MG_CLUSTER_2.html]

Details for gene set DOROSZKO\_U3180MG\_CLUSTER\_2[GSEA]

|  || Dataset | state1 |
| Phenotype | NoPhenotypeAvailable |
| Upregulated in class | na\_neg |
| GeneSet | DOROSZKO\_U3180MG\_CLUSTER\_2 |
| Enrichment Score (ES) | -0.7050805 |
| Normalized Enrichment Score (NES) | -2.9648747 |
| Nominal p-value | 0.0 |
| FDR q-value | 0.0 |
| FWER p-Value | 0.0 |
Table: GSEA Results Summary

  

Fig 1: Enrichment plot: DOROSZKO\_U3180MG\_CLUSTER\_2      
 Profile of the Running ES Score & Positions of GeneSet Members on the Rank Ordered List

  

| PROBE | GENE SYMBOL | GENE\_TITLE | RANK IN GENE LIST | RANK METRIC SCORE | RUNNING ES | CORE ENRICHMENT || 1 | LMNA |  |  | 9 | 0.701 | 0.0082 | No |
| 2 | IGFBP3 |  |  | 15 | 0.664 | 0.0164 | No |
| 3 | TNFRSF12A |  |  | 104 | 0.364 | 0.0119 | No |
| 4 | YBX3 |  |  | 163 | 0.314 | 0.0098 | No |
| 5 | HSPD1 |  |  | 397 | 0.227 | -0.0120 | No |
| 6 | GOLIM4 |  |  | 525 | 0.200 | -0.0228 | No |
| 7 | PPP1CB |  |  | 534 | 0.198 | -0.0211 | No |
| 8 | IDS |  |  | 625 | 0.182 | -0.0282 | No |
| 9 | EMD |  |  | 687 | 0.174 | -0.0324 | No |
| 10 | CAMK2D |  |  | 689 | 0.174 | -0.0303 | No |
| 11 | IFRD2 |  |  | 713 | 0.170 | -0.0305 | No |
| 12 | PGP |  |  | 852 | 0.151 | -0.0431 | No |
| 13 | CNTN1 |  |  | 911 | 0.143 | -0.0474 | No |
| 14 | CALD1 |  |  | 968 | 0.137 | -0.0516 | No |
| 15 | NTM |  |  | 1002 | 0.134 | -0.0533 | No |
| 16 | GADD45A |  |  | 1082 | 0.124 | -0.0601 | No |
| 17 | KTN1 |  |  | 1113 | 0.121 | -0.0617 | No |
| 18 | FERMT1 |  |  | 1163 | 0.117 | -0.0653 | No |
| 19 | IAH1 |  |  | 1206 | 0.112 | -0.0683 | No |
| 20 | MEIS2 |  |  | 1230 | 0.110 | -0.0693 | No |
| 21 | CEBPZOS |  |  | 1248 | 0.108 | -0.0697 | No |
| 22 | GNL3 |  |  | 1256 | 0.108 | -0.0691 | No |
| 23 | COL6A1 |  |  | 1317 | 0.102 | -0.0741 | No |
| 24 | ARRDC3 |  |  | 1347 | 0.099 | -0.0759 | No |
| 25 | DNPEP |  |  | 1349 | 0.099 | -0.0747 | No |
| 26 | CTTN |  |  | 1411 | 0.095 | -0.0799 | No |
| 27 | SLC3A2 |  |  | 1646 | 0.079 | -0.1037 | No |
| 28 | LIMA1 |  |  | 1678 | 0.077 | -0.1060 | No |
| 29 | ARHGAP21 |  |  | 1748 | 0.072 | -0.1124 | No |
| 30 | CEBPZ |  |  | 1753 | 0.072 | -0.1119 | No |
| 31 | ABCE1 |  |  | 1790 | 0.070 | -0.1148 | No |
| 32 | MTRF1L |  |  | 1804 | 0.069 | -0.1152 | No |
| 33 | TDG |  |  | 1945 | 0.063 | -0.1293 | No |
| 34 | DDIT3 |  |  | 2008 | 0.059 | -0.1351 | No |
| 35 | FAM120A |  |  | 2038 | 0.058 | -0.1374 | No |
| 36 | CYR61 |  |  | 2172 | 0.052 | -0.1509 | No |
| 37 | HSPH1 |  |  | 2441 | 0.042 | -0.1788 | No |
| 38 | PTBP1 |  |  | 2448 | 0.041 | -0.1789 | No |
| 39 | PSMC6 |  |  | 2532 | 0.038 | -0.1872 | No |
| 40 | HERPUD1 |  |  | 2598 | 0.036 | -0.1936 | No |
| 41 | TIMM44 |  |  | 2908 | 0.027 | -0.2261 | No |
| 42 | CFLAR |  |  | 2958 | 0.026 | -0.2309 | No |
| 43 | USP16 |  |  | 3016 | 0.025 | -0.2367 | No |
| 44 | TLK1 |  |  | 3089 | 0.023 | -0.2440 | No |
| 45 | SNHG9 |  |  | 3217 | 0.020 | -0.2572 | No |
| 46 | DDX3X |  |  | 3240 | 0.020 | -0.2593 | No |
| 47 | TUBGCP2 |  |  | 3297 | 0.018 | -0.2650 | No |
| 48 | DST |  |  | 3373 | 0.017 | -0.2728 | No |
| 49 | CDC37 |  |  | 3539 | 0.013 | -0.2901 | No |
| 50 | DNAJC2 |  |  | 3593 | 0.012 | -0.2956 | No |
| 51 | DDX42 |  |  | 3634 | 0.011 | -0.2997 | No |
| 52 | FASTK |  |  | 3665 | 0.011 | -0.3028 | No |
| 53 | AHCYL1 |  |  | 3686 | 0.010 | -0.3047 | No |
| 54 | HSPA4 |  |  | 3742 | 0.009 | -0.3105 | No |
| 55 | SRSF2 |  |  | 3783 | 0.008 | -0.3146 | No |
| 56 | ITGB1 |  |  | 3956 | 0.005 | -0.3328 | No |
| 57 | MYC |  |  | 3964 | 0.005 | -0.3335 | No |
| 58 | ABHD12 |  |  | 3974 | 0.005 | -0.3344 | No |
| 59 | ELMSAN1 |  |  | 3997 | 0.004 | -0.3367 | No |
| 60 | YME1L1 |  |  | 4055 | 0.003 | -0.3427 | No |
| 61 | SACS |  |  | 4144 | 0.002 | -0.3520 | No |
| 62 | DPF3 |  |  | 4225 | 0.000 | -0.3605 | No |
| 63 | EWSR1 |  |  | 4279 | -0.001 | -0.3661 | No |
| 64 | HSPA1B |  |  | 4282 | -0.001 | -0.3663 | No |
| 65 | SRSF11 |  |  | 4379 | -0.002 | -0.3765 | No |
| 66 | WEE1 |  |  | 4385 | -0.003 | -0.3770 | No |
| 67 | FOXN2 |  |  | 4398 | -0.003 | -0.3782 | No |
| 68 | SRSF7 |  |  | 4413 | -0.003 | -0.3797 | No |
| 69 | ACADVL |  |  | 4425 | -0.003 | -0.3808 | No |
| 70 | SUPT20H |  |  | 4496 | -0.004 | -0.3882 | No |
| 71 | ARIH2 |  |  | 4585 | -0.006 | -0.3974 | No |
| 72 | TCERG1 |  |  | 4597 | -0.006 | -0.3985 | No |
| 73 | TMEM120A |  |  | 4741 | -0.008 | -0.4136 | No |
| 74 | DMAP1 |  |  | 4800 | -0.009 | -0.4196 | No |
| 75 | ATXN7L3B |  |  | 4972 | -0.012 | -0.4376 | No |
| 76 | RNF115 |  |  | 4991 | -0.013 | -0.4394 | No |
| 77 | CHMP4A |  |  | 5038 | -0.013 | -0.4441 | No |
| 78 | PCMT1 |  |  | 5043 | -0.013 | -0.4444 | No |
| 79 | NAA15 |  |  | 5074 | -0.014 | -0.4474 | No |
| 80 | ANAPC5 |  |  | 5107 | -0.014 | -0.4506 | No |
| 81 | PRRC2A |  |  | 5142 | -0.015 | -0.4540 | No |
| 82 | MTA1 |  |  | 5162 | -0.015 | -0.4558 | No |
| 83 | SAFB2 |  |  | 5227 | -0.016 | -0.4624 | No |
| 84 | TUFM |  |  | 5247 | -0.016 | -0.4642 | No |
| 85 | TRA2B |  |  | 5321 | -0.017 | -0.4717 | No |
| 86 | ARHGAP5 |  |  | 5327 | -0.017 | -0.4721 | No |
| 87 | USP3 |  |  | 5375 | -0.018 | -0.4768 | No |
| 88 | HAGHL |  |  | 5467 | -0.020 | -0.4862 | No |
| 89 | NCOR1 |  |  | 5490 | -0.020 | -0.4883 | No |
| 90 | XBP1 |  |  | 5510 | -0.020 | -0.4900 | No |
| 91 | TLE4 |  |  | 5578 | -0.021 | -0.4969 | No |
| 92 | LUC7L |  |  | 5616 | -0.022 | -0.5005 | No |
| 93 | ANAPC7 |  |  | 5663 | -0.023 | -0.5051 | No |
| 94 | ANK2 |  |  | 5675 | -0.023 | -0.5060 | No |
| 95 | MYCBP2 |  |  | 5769 | -0.025 | -0.5155 | No |
| 96 | VPS37B |  |  | 5829 | -0.026 | -0.5215 | No |
| 97 | SFXN5 |  |  | 5908 | -0.028 | -0.5294 | No |
| 98 | CWC25 |  |  | 5981 | -0.029 | -0.5366 | No |
| 99 | DTNA |  |  | 6001 | -0.029 | -0.5383 | No |
| 100 | SFPQ |  |  | 6115 | -0.031 | -0.5499 | No |
| 101 | NOP56 |  |  | 6205 | -0.033 | -0.5589 | No |
| 102 | DNM1L |  |  | 6273 | -0.034 | -0.5656 | No |
| 103 | HMGXB4 |  |  | 6279 | -0.034 | -0.5656 | No |
| 104 | BCLAF1 |  |  | 6289 | -0.034 | -0.5661 | No |
| 105 | DTX3 |  |  | 6298 | -0.035 | -0.5665 | No |
| 106 | GDAP1 |  |  | 6306 | -0.035 | -0.5668 | No |
| 107 | ABHD5 |  |  | 6384 | -0.036 | -0.5745 | No |
| 108 | DDX46 |  |  | 6456 | -0.038 | -0.5816 | No |
| 109 | OSBPL1A |  |  | 6495 | -0.038 | -0.5851 | No |
| 110 | ORAI2 |  |  | 6535 | -0.039 | -0.5887 | No |
| 111 | PHF20 |  |  | 6556 | -0.040 | -0.5903 | No |
| 112 | GNPAT |  |  | 6663 | -0.042 | -0.6010 | No |
| 113 | MED6 |  |  | 6730 | -0.044 | -0.6075 | No |
| 114 | RBM23 |  |  | 6744 | -0.044 | -0.6083 | No |
| 115 | NCK1 |  |  | 6783 | -0.045 | -0.6117 | No |
| 116 | LRP10 |  |  | 6814 | -0.045 | -0.6143 | No |
| 117 | DDB1 |  |  | 6857 | -0.046 | -0.6182 | No |
| 118 | RBM28 |  |  | 6863 | -0.046 | -0.6181 | No |
| 119 | ATP6V0B |  |  | 6884 | -0.047 | -0.6196 | No |
| 120 | SRSF1 |  |  | 6896 | -0.047 | -0.6202 | No |
| 121 | CNOT2 |  |  | 6899 | -0.047 | -0.6198 | No |
| 122 | ARFGAP2 |  |  | 6918 | -0.048 | -0.6210 | No |
| 123 | CRTC3 |  |  | 6937 | -0.048 | -0.6223 | No |
| 124 | SMCHD1 |  |  | 6939 | -0.048 | -0.6218 | No |
| 125 | ZRANB2 |  |  | 6969 | -0.049 | -0.6242 | No |
| 126 | THAP5 |  |  | 6999 | -0.050 | -0.6266 | No |
| 127 | IP6K2 |  |  | 7026 | -0.051 | -0.6287 | No |
| 128 | SRSF6 |  |  | 7028 | -0.051 | -0.6282 | No |
| 129 | PRPF4B |  |  | 7096 | -0.053 | -0.6346 | No |
| 130 | COMMD2 |  |  | 7125 | -0.054 | -0.6369 | No |
| 131 | B3GAT3 |  |  | 7146 | -0.054 | -0.6383 | No |
| 132 | SLC12A9 |  |  | 7147 | -0.054 | -0.6376 | No |
| 133 | IST1 |  |  | 7162 | -0.054 | -0.6383 | No |
| 134 | PTK2 |  |  | 7170 | -0.055 | -0.6384 | No |
| 135 | BDP1 |  |  | 7209 | -0.056 | -0.6417 | No |
| 136 | ARHGEF2 |  |  | 7210 | -0.056 | -0.6409 | No |
| 137 | LONP2 |  |  | 7238 | -0.056 | -0.6431 | No |
| 138 | TRIM44 |  |  | 7279 | -0.057 | -0.6466 | No |
| 139 | TRMT13 |  |  | 7287 | -0.058 | -0.6466 | No |
| 140 | PBXIP1 |  |  | 7302 | -0.058 | -0.6473 | No |
| 141 | RPRD1A |  |  | 7345 | -0.059 | -0.6510 | No |
| 142 | IFRD1 |  |  | 7353 | -0.059 | -0.6509 | No |
| 143 | STX16 |  |  | 7367 | -0.060 | -0.6515 | No |
| 144 | ST5 |  |  | 7393 | -0.060 | -0.6534 | No |
| 145 | NELFCD |  |  | 7396 | -0.061 | -0.6528 | No |
| 146 | CLK1 |  |  | 7413 | -0.061 | -0.6537 | No |
| 147 | JAG1 |  |  | 7426 | -0.061 | -0.6542 | No |
| 148 | SERPINH1 |  |  | 7462 | -0.062 | -0.6571 | No |
| 149 | NOC2L |  |  | 7468 | -0.062 | -0.6568 | No |
| 150 | FAM172A |  |  | 7474 | -0.062 | -0.6565 | No |
| 151 | MLF1 |  |  | 7491 | -0.063 | -0.6574 | No |
| 152 | PRPF38B |  |  | 7500 | -0.063 | -0.6574 | No |
| 153 | SRSF10 |  |  | 7557 | -0.065 | -0.6625 | No |
| 154 | RB1CC1 |  |  | 7558 | -0.065 | -0.6616 | No |
| 155 | STAT3 |  |  | 7597 | -0.066 | -0.6648 | No |
| 156 | MPHOSPH8 |  |  | 7604 | -0.066 | -0.6646 | No |
| 157 | PRPSAP1 |  |  | 7643 | -0.067 | -0.6677 | No |
| 158 | NSUN5 |  |  | 7670 | -0.069 | -0.6696 | No |
| 159 | SPSB3 |  |  | 7673 | -0.069 | -0.6689 | No |
| 160 | CTSL |  |  | 7676 | -0.069 | -0.6682 | No |
| 161 | PPP1R16A |  |  | 7703 | -0.070 | -0.6700 | No |
| 162 | NPDC1 |  |  | 7756 | -0.072 | -0.6746 | No |
| 163 | LFNG |  |  | 7784 | -0.073 | -0.6765 | No |
| 164 | DPF2 |  |  | 7789 | -0.073 | -0.6760 | No |
| 165 | GPAA1 |  |  | 7813 | -0.074 | -0.6775 | No |
| 166 | USP33 |  |  | 7817 | -0.074 | -0.6768 | No |
| 167 | RIMKLB |  |  | 7846 | -0.075 | -0.6788 | No |
| 168 | QTRT1 |  |  | 7874 | -0.076 | -0.6807 | No |
| 169 | CDC42BPA |  |  | 7878 | -0.077 | -0.6800 | No |
| 170 | PBRM1 |  |  | 7915 | -0.078 | -0.6828 | No |
| 171 | HECTD1 |  |  | 7932 | -0.079 | -0.6835 | No |
| 172 | USP34 |  |  | 7937 | -0.079 | -0.6829 | No |
| 173 | U2SURP |  |  | 7943 | -0.079 | -0.6824 | No |
| 174 | CCS |  |  | 7948 | -0.079 | -0.6817 | No |
| 175 | DHX30 |  |  | 7961 | -0.080 | -0.6820 | No |
| 176 | TCF25 |  |  | 7997 | -0.081 | -0.6846 | No |
| 177 | HNRNPH1 |  |  | 8004 | -0.081 | -0.6842 | No |
| 178 | MCL1 |  |  | 8011 | -0.082 | -0.6838 | No |
| 179 | TIA1 |  |  | 8017 | -0.082 | -0.6832 | No |
| 180 | SECISBP2 |  |  | 8024 | -0.082 | -0.6828 | No |
| 181 | AC020916.1 |  |  | 8032 | -0.082 | -0.6824 | No |
| 182 | FJX1 |  |  | 8037 | -0.083 | -0.6818 | No |
| 183 | SRSF5 |  |  | 8049 | -0.083 | -0.6818 | No |
| 184 | PPIB |  |  | 8099 | -0.085 | -0.6859 | No |
| 185 | RBM4B |  |  | 8109 | -0.086 | -0.6858 | No |
| 186 | CAND1 |  |  | 8150 | -0.087 | -0.6889 | No |
| 187 | ZNF655 |  |  | 8154 | -0.087 | -0.6880 | No |
| 188 | SNRNP200 |  |  | 8189 | -0.089 | -0.6905 | No |
| 189 | KMT2A |  |  | 8304 | -0.095 | -0.7014 | No |
| 190 | APOE |  |  | 8322 | -0.095 | -0.7019 | No |
| 191 | CHD1 |  |  | 8336 | -0.096 | -0.7020 | No |
| 192 | KNOP1 |  |  | 8340 | -0.096 | -0.7011 | No |
| 193 | LARS |  |  | 8345 | -0.096 | -0.7002 | No |
| 194 | TTC37 |  |  | 8349 | -0.097 | -0.6993 | No |
| 195 | BSG |  |  | 8399 | -0.099 | -0.7032 | No |
| 196 | CTSA |  |  | 8404 | -0.099 | -0.7023 | No |
| 197 | ZNF680 |  |  | 8408 | -0.100 | -0.7013 | No |
| 198 | RAF1 |  |  | 8419 | -0.100 | -0.7011 | No |
| 199 | MACF1 |  |  | 8426 | -0.100 | -0.7004 | No |
| 200 | RNF170 |  |  | 8471 | -0.103 | -0.7037 | Yes |
| 201 | RNMT |  |  | 8472 | -0.103 | -0.7024 | Yes |
| 202 | EIF2S3 |  |  | 8476 | -0.103 | -0.7013 | Yes |
| 203 | TMUB2 |  |  | 8501 | -0.105 | -0.7025 | Yes |
| 204 | TMEM87A |  |  | 8511 | -0.106 | -0.7021 | Yes |
| 205 | CCDC66 |  |  | 8512 | -0.106 | -0.7007 | Yes |
| 206 | ZNF207 |  |  | 8534 | -0.107 | -0.7015 | Yes |
| 207 | TERF1 |  |  | 8543 | -0.108 | -0.7009 | Yes |
| 208 | AFF4 |  |  | 8548 | -0.108 | -0.6999 | Yes |
| 209 | RCN1 |  |  | 8553 | -0.108 | -0.6989 | Yes |
| 210 | TRA2A |  |  | 8564 | -0.109 | -0.6986 | Yes |
| 211 | CYHR1 |  |  | 8568 | -0.110 | -0.6975 | Yes |
| 212 | NCSTN |  |  | 8576 | -0.110 | -0.6968 | Yes |
| 213 | MTF2 |  |  | 8595 | -0.111 | -0.6972 | Yes |
| 214 | ZNF131 |  |  | 8609 | -0.112 | -0.6971 | Yes |
| 215 | RRBP1 |  |  | 8613 | -0.112 | -0.6960 | Yes |
| 216 | HNRNPU |  |  | 8616 | -0.112 | -0.6947 | Yes |
| 217 | GPATCH2 |  |  | 8645 | -0.114 | -0.6962 | Yes |
| 218 | PKN2 |  |  | 8647 | -0.114 | -0.6948 | Yes |
| 219 | RPAP2 |  |  | 8668 | -0.115 | -0.6954 | Yes |
| 220 | DDX18 |  |  | 8676 | -0.116 | -0.6946 | Yes |
| 221 | KMT5B |  |  | 8680 | -0.116 | -0.6934 | Yes |
| 222 | IFNGR1 |  |  | 8689 | -0.116 | -0.6927 | Yes |
| 223 | DAAM1 |  |  | 8691 | -0.117 | -0.6913 | Yes |
| 224 | SNRNP70 |  |  | 8708 | -0.118 | -0.6915 | Yes |
| 225 | CCDC82 |  |  | 8711 | -0.118 | -0.6901 | Yes |
| 226 | CSNK2A1 |  |  | 8724 | -0.119 | -0.6898 | Yes |
| 227 | MAT2A |  |  | 8741 | -0.121 | -0.6899 | Yes |
| 228 | NEMF |  |  | 8755 | -0.122 | -0.6897 | Yes |
| 229 | PTCD3 |  |  | 8758 | -0.122 | -0.6883 | Yes |
| 230 | CELF1 |  |  | 8768 | -0.123 | -0.6877 | Yes |
| 231 | M6PR |  |  | 8772 | -0.123 | -0.6864 | Yes |
| 232 | SAR1B |  |  | 8778 | -0.123 | -0.6853 | Yes |
| 233 | MYO6 |  |  | 8788 | -0.124 | -0.6846 | Yes |
| 234 | GTF2H2 |  |  | 8791 | -0.124 | -0.6832 | Yes |
| 235 | ZNF800 |  |  | 8792 | -0.124 | -0.6816 | Yes |
| 236 | GCC2 |  |  | 8799 | -0.124 | -0.6806 | Yes |
| 237 | SLC39A1 |  |  | 8800 | -0.124 | -0.6789 | Yes |
| 238 | COPA |  |  | 8814 | -0.126 | -0.6787 | Yes |
| 239 | RBM25 |  |  | 8828 | -0.127 | -0.6784 | Yes |
| 240 | TTC19 |  |  | 8831 | -0.127 | -0.6769 | Yes |
| 241 | RIF1 |  |  | 8837 | -0.128 | -0.6758 | Yes |
| 242 | HOOK3 |  |  | 8838 | -0.128 | -0.6741 | Yes |
| 243 | CPEB4 |  |  | 8849 | -0.128 | -0.6735 | Yes |
| 244 | THOC2 |  |  | 8859 | -0.129 | -0.6728 | Yes |
| 245 | DDX6 |  |  | 8860 | -0.130 | -0.6711 | Yes |
| 246 | ARID4A |  |  | 8864 | -0.130 | -0.6697 | Yes |
| 247 | SELENOS |  |  | 8867 | -0.130 | -0.6682 | Yes |
| 248 | SUMF2 |  |  | 8893 | -0.134 | -0.6691 | Yes |
| 249 | DHX36 |  |  | 8895 | -0.134 | -0.6674 | Yes |
| 250 | DDX5 |  |  | 8901 | -0.134 | -0.6662 | Yes |
| 251 | CNOT4 |  |  | 8916 | -0.136 | -0.6659 | Yes |
| 252 | SLC25A36 |  |  | 8918 | -0.136 | -0.6642 | Yes |
| 253 | AUP1 |  |  | 8957 | -0.139 | -0.6665 | Yes |
| 254 | FAM53C |  |  | 8962 | -0.140 | -0.6650 | Yes |
| 255 | PUS7L |  |  | 8967 | -0.140 | -0.6636 | Yes |
| 256 | PSRC1 |  |  | 8973 | -0.141 | -0.6623 | Yes |
| 257 | AKAP9 |  |  | 8975 | -0.141 | -0.6606 | Yes |
| 258 | FSTL1 |  |  | 8976 | -0.141 | -0.6587 | Yes |
| 259 | ADAM9 |  |  | 8977 | -0.141 | -0.6569 | Yes |
| 260 | TMEM59L |  |  | 8978 | -0.141 | -0.6550 | Yes |
| 261 | P4HTM |  |  | 8982 | -0.141 | -0.6535 | Yes |
| 262 | RBM39 |  |  | 8983 | -0.141 | -0.6516 | Yes |
| 263 | RSBN1L |  |  | 8987 | -0.142 | -0.6501 | Yes |
| 264 | WDR60 |  |  | 8989 | -0.142 | -0.6483 | Yes |
| 265 | TAOK1 |  |  | 8990 | -0.142 | -0.6465 | Yes |
| 266 | UBR5 |  |  | 8995 | -0.142 | -0.6450 | Yes |
| 267 | RIC3 |  |  | 9004 | -0.143 | -0.6440 | Yes |
| 268 | TMEM30A |  |  | 9008 | -0.143 | -0.6425 | Yes |
| 269 | NIN |  |  | 9011 | -0.143 | -0.6408 | Yes |
| 270 | ESF1 |  |  | 9013 | -0.143 | -0.6390 | Yes |
| 271 | NSD1 |  |  | 9020 | -0.144 | -0.6378 | Yes |
| 272 | COLGALT2 |  |  | 9025 | -0.144 | -0.6363 | Yes |
| 273 | JPX |  |  | 9034 | -0.145 | -0.6352 | Yes |
| 274 | PCM1 |  |  | 9037 | -0.146 | -0.6335 | Yes |
| 275 | AIG1 |  |  | 9046 | -0.147 | -0.6325 | Yes |
| 276 | MARK3 |  |  | 9052 | -0.147 | -0.6310 | Yes |
| 277 | SON |  |  | 9059 | -0.148 | -0.6297 | Yes |
| 278 | SLC25A37 |  |  | 9067 | -0.149 | -0.6285 | Yes |
| 279 | LMAN1 |  |  | 9094 | -0.151 | -0.6293 | Yes |
| 280 | TAF1D |  |  | 9096 | -0.152 | -0.6274 | Yes |
| 281 | HM13 |  |  | 9098 | -0.152 | -0.6255 | Yes |
| 282 | SPPL2A |  |  | 9100 | -0.152 | -0.6236 | Yes |
| 283 | SPTLC1 |  |  | 9103 | -0.152 | -0.6219 | Yes |
| 284 | ARHGEF7 |  |  | 9108 | -0.153 | -0.6203 | Yes |
| 285 | KIF21A |  |  | 9110 | -0.153 | -0.6184 | Yes |
| 286 | CD164 |  |  | 9121 | -0.154 | -0.6174 | Yes |
| 287 | ZNF37A |  |  | 9125 | -0.154 | -0.6157 | Yes |
| 288 | SREK1 |  |  | 9144 | -0.157 | -0.6156 | Yes |
| 289 | VCAN |  |  | 9151 | -0.158 | -0.6141 | Yes |
| 290 | WDR73 |  |  | 9153 | -0.159 | -0.6122 | Yes |
| 291 | FGFR1 |  |  | 9157 | -0.159 | -0.6104 | Yes |
| 292 | GPATCH8 |  |  | 9159 | -0.160 | -0.6084 | Yes |
| 293 | RBM26 |  |  | 9161 | -0.160 | -0.6064 | Yes |
| 294 | GUSB |  |  | 9165 | -0.161 | -0.6046 | Yes |
| 295 | RAB3IP |  |  | 9166 | -0.161 | -0.6025 | Yes |
| 296 | SF3B1 |  |  | 9177 | -0.162 | -0.6014 | Yes |
| 297 | NPAS3 |  |  | 9181 | -0.163 | -0.5996 | Yes |
| 298 | UNC50 |  |  | 9182 | -0.163 | -0.5975 | Yes |
| 299 | CDK5RAP3 |  |  | 9183 | -0.163 | -0.5953 | Yes |
| 300 | CDH2 |  |  | 9187 | -0.163 | -0.5935 | Yes |
| 301 | SMARCC2 |  |  | 9188 | -0.163 | -0.5914 | Yes |
| 302 | ANKRD12 |  |  | 9194 | -0.165 | -0.5897 | Yes |
| 303 | SPTAN1 |  |  | 9199 | -0.165 | -0.5880 | Yes |
| 304 | EXOC7 |  |  | 9213 | -0.167 | -0.5872 | Yes |
| 305 | PRRC2B |  |  | 9217 | -0.168 | -0.5853 | Yes |
| 306 | ASH1L |  |  | 9227 | -0.169 | -0.5840 | Yes |
| 307 | KMT2C |  |  | 9233 | -0.170 | -0.5823 | Yes |
| 308 | GON4L |  |  | 9236 | -0.171 | -0.5803 | Yes |
| 309 | EPN2 |  |  | 9249 | -0.172 | -0.5793 | Yes |
| 310 | JMJD1C |  |  | 9250 | -0.172 | -0.5770 | Yes |
| 311 | PJA2 |  |  | 9253 | -0.173 | -0.5750 | Yes |
| 312 | ADAR |  |  | 9255 | -0.173 | -0.5728 | Yes |
| 313 | MFSD10 |  |  | 9258 | -0.173 | -0.5708 | Yes |
| 314 | FAM133B |  |  | 9263 | -0.174 | -0.5689 | Yes |
| 315 | SAT2 |  |  | 9272 | -0.175 | -0.5675 | Yes |
| 316 | HES1 |  |  | 9280 | -0.177 | -0.5659 | Yes |
| 317 | FNIP1 |  |  | 9281 | -0.177 | -0.5636 | Yes |
| 318 | CTSD |  |  | 9287 | -0.178 | -0.5617 | Yes |
| 319 | PHF14 |  |  | 9289 | -0.178 | -0.5595 | Yes |
| 320 | CHD6 |  |  | 9290 | -0.178 | -0.5572 | Yes |
| 321 | CTNNB1 |  |  | 9294 | -0.179 | -0.5551 | Yes |
| 322 | KMT2E |  |  | 9297 | -0.180 | -0.5530 | Yes |
| 323 | DDRGK1 |  |  | 9309 | -0.183 | -0.5518 | Yes |
| 324 | B4GAT1 |  |  | 9313 | -0.183 | -0.5497 | Yes |
| 325 | EXOC4 |  |  | 9315 | -0.183 | -0.5474 | Yes |
| 326 | ABI2 |  |  | 9319 | -0.184 | -0.5453 | Yes |
| 327 | GATAD1 |  |  | 9325 | -0.186 | -0.5434 | Yes |
| 328 | ACSL3 |  |  | 9338 | -0.187 | -0.5422 | Yes |
| 329 | TBL1XR1 |  |  | 9339 | -0.187 | -0.5397 | Yes |
| 330 | SEC61A1 |  |  | 9343 | -0.188 | -0.5376 | Yes |
| 331 | MPZL1 |  |  | 9345 | -0.189 | -0.5352 | Yes |
| 332 | CCNL1 |  |  | 9347 | -0.189 | -0.5328 | Yes |
| 333 | ZNF302 |  |  | 9349 | -0.190 | -0.5304 | Yes |
| 334 | TAOK3 |  |  | 9351 | -0.190 | -0.5281 | Yes |
| 335 | OS9 |  |  | 9355 | -0.191 | -0.5259 | Yes |
| 336 | SLC22A17 |  |  | 9357 | -0.191 | -0.5235 | Yes |
| 337 | SYNE2 |  |  | 9362 | -0.193 | -0.5214 | Yes |
| 338 | HSP90B1 |  |  | 9365 | -0.193 | -0.5190 | Yes |
| 339 | GPBP1 |  |  | 9371 | -0.194 | -0.5170 | Yes |
| 340 | TM9SF2 |  |  | 9374 | -0.195 | -0.5147 | Yes |
| 341 | SPG7 |  |  | 9380 | -0.196 | -0.5126 | Yes |
| 342 | CCDC14 |  |  | 9388 | -0.198 | -0.5108 | Yes |
| 343 | ZNF292 |  |  | 9395 | -0.200 | -0.5088 | Yes |
| 344 | PRKCSH |  |  | 9399 | -0.200 | -0.5065 | Yes |
| 345 | GALR1 |  |  | 9404 | -0.201 | -0.5043 | Yes |
| 346 | PABPN1 |  |  | 9405 | -0.201 | -0.5016 | Yes |
| 347 | BAZ2B |  |  | 9413 | -0.204 | -0.4997 | Yes |
| 348 | POR |  |  | 9415 | -0.204 | -0.4971 | Yes |
| 349 | ATF6B |  |  | 9416 | -0.204 | -0.4944 | Yes |
| 350 | OFD1 |  |  | 9417 | -0.205 | -0.4917 | Yes |
| 351 | BICD1 |  |  | 9419 | -0.205 | -0.4892 | Yes |
| 352 | ASPH |  |  | 9421 | -0.205 | -0.4866 | Yes |
| 353 | RDH11 |  |  | 9422 | -0.205 | -0.4839 | Yes |
| 354 | REV3L |  |  | 9431 | -0.208 | -0.4820 | Yes |
| 355 | GOLGB1 |  |  | 9435 | -0.208 | -0.4796 | Yes |
| 356 | NASP |  |  | 9437 | -0.208 | -0.4770 | Yes |
| 357 | GPRC5B |  |  | 9454 | -0.214 | -0.4759 | Yes |
| 358 | FKBP10 |  |  | 9455 | -0.215 | -0.4731 | Yes |
| 359 | NAV1 |  |  | 9460 | -0.216 | -0.4706 | Yes |
| 360 | DDOST |  |  | 9461 | -0.217 | -0.4678 | Yes |
| 361 | ZNF638 |  |  | 9464 | -0.217 | -0.4651 | Yes |
| 362 | MKLN1 |  |  | 9467 | -0.218 | -0.4625 | Yes |
| 363 | SPECC1 |  |  | 9470 | -0.219 | -0.4598 | Yes |
| 364 | MUM1 |  |  | 9477 | -0.220 | -0.4576 | Yes |
| 365 | SPAG9 |  |  | 9487 | -0.223 | -0.4556 | Yes |
| 366 | MSI2 |  |  | 9498 | -0.225 | -0.4537 | Yes |
| 367 | FOXO3 |  |  | 9503 | -0.226 | -0.4512 | Yes |
| 368 | CLSTN1 |  |  | 9504 | -0.227 | -0.4482 | Yes |
| 369 | SLC16A1 |  |  | 9509 | -0.227 | -0.4456 | Yes |
| 370 | PDIA4 |  |  | 9513 | -0.229 | -0.4430 | Yes |
| 371 | PNN |  |  | 9517 | -0.230 | -0.4403 | Yes |
| 372 | SERINC1 |  |  | 9530 | -0.233 | -0.4385 | Yes |
| 373 | HSPA5 |  |  | 9533 | -0.234 | -0.4356 | Yes |
| 374 | ZNF397 |  |  | 9534 | -0.234 | -0.4325 | Yes |
| 375 | CALR |  |  | 9537 | -0.235 | -0.4297 | Yes |
| 376 | ACAP3 |  |  | 9544 | -0.237 | -0.4272 | Yes |
| 377 | PLPP3 |  |  | 9549 | -0.239 | -0.4245 | Yes |
| 378 | LAPTM4A |  |  | 9553 | -0.241 | -0.4216 | Yes |
| 379 | HEPACAM |  |  | 9555 | -0.242 | -0.4186 | Yes |
| 380 | TMEM165 |  |  | 9559 | -0.244 | -0.4157 | Yes |
| 381 | CTSF |  |  | 9563 | -0.246 | -0.4128 | Yes |
| 382 | PRKCA |  |  | 9569 | -0.248 | -0.4101 | Yes |
| 383 | MAGI1 |  |  | 9576 | -0.250 | -0.4074 | Yes |
| 384 | TTC14 |  |  | 9578 | -0.252 | -0.4042 | Yes |
| 385 | ATP6AP1 |  |  | 9582 | -0.254 | -0.4012 | Yes |
| 386 | EPM2AIP1 |  |  | 9585 | -0.255 | -0.3981 | Yes |
| 387 | CHD9 |  |  | 9587 | -0.255 | -0.3948 | Yes |
| 388 | TNRC6B |  |  | 9589 | -0.256 | -0.3916 | Yes |
| 389 | GRN |  |  | 9593 | -0.257 | -0.3885 | Yes |
| 390 | LAMP1 |  |  | 9594 | -0.257 | -0.3852 | Yes |
| 391 | TNC |  |  | 9605 | -0.262 | -0.3828 | Yes |
| 392 | DPP6 |  |  | 9606 | -0.262 | -0.3793 | Yes |
| 393 | GTF2I |  |  | 9607 | -0.262 | -0.3759 | Yes |
| 394 | SPTBN1 |  |  | 9608 | -0.263 | -0.3725 | Yes |
| 395 | ATP2A2 |  |  | 9610 | -0.263 | -0.3691 | Yes |
| 396 | CREB5 |  |  | 9616 | -0.265 | -0.3662 | Yes |
| 397 | PNISR |  |  | 9627 | -0.272 | -0.3637 | Yes |
| 398 | DYNC1H1 |  |  | 9632 | -0.273 | -0.3605 | Yes |
| 399 | IRS2 |  |  | 9641 | -0.277 | -0.3577 | Yes |
| 400 | RGS12 |  |  | 9644 | -0.277 | -0.3543 | Yes |
| 401 | KIF1B |  |  | 9649 | -0.282 | -0.3510 | Yes |
| 402 | ITGAV |  |  | 9650 | -0.282 | -0.3473 | Yes |
| 403 | RAB6A |  |  | 9652 | -0.283 | -0.3437 | Yes |
| 404 | ATRX |  |  | 9654 | -0.284 | -0.3401 | Yes |
| 405 | RFX4 |  |  | 9655 | -0.285 | -0.3363 | Yes |
| 406 | RTN4 |  |  | 9661 | -0.288 | -0.3331 | Yes |
| 407 | SETD5 |  |  | 9663 | -0.291 | -0.3294 | Yes |
| 408 | CCDC47 |  |  | 9664 | -0.291 | -0.3256 | Yes |
| 409 | MFGE8 |  |  | 9665 | -0.291 | -0.3217 | Yes |
| 410 | LUC7L3 |  |  | 9667 | -0.292 | -0.3180 | Yes |
| 411 | DLL3 |  |  | 9678 | -0.297 | -0.3152 | Yes |
| 412 | CCDC88A |  |  | 9679 | -0.298 | -0.3113 | Yes |
| 413 | PODXL2 |  |  | 9686 | -0.301 | -0.3080 | Yes |
| 414 | PDIA3 |  |  | 9687 | -0.301 | -0.3040 | Yes |
| 415 | LRPAP1 |  |  | 9691 | -0.305 | -0.3003 | Yes |
| 416 | RSRP1 |  |  | 9694 | -0.309 | -0.2965 | Yes |
| 417 | GLG1 |  |  | 9698 | -0.313 | -0.2927 | Yes |
| 418 | PDIA6 |  |  | 9701 | -0.314 | -0.2888 | Yes |
| 419 | N4BP2L2 |  |  | 9712 | -0.326 | -0.2856 | Yes |
| 420 | SLC35F1 |  |  | 9715 | -0.327 | -0.2815 | Yes |
| 421 | DCLK2 |  |  | 9717 | -0.328 | -0.2773 | Yes |
| 422 | SF1 |  |  | 9735 | -0.344 | -0.2746 | Yes |
| 423 | APLP2 |  |  | 9736 | -0.344 | -0.2701 | Yes |
| 424 | PSAP |  |  | 9742 | -0.351 | -0.2660 | Yes |
| 425 | ZBTB20 |  |  | 9743 | -0.352 | -0.2614 | Yes |
| 426 | ZEB2 |  |  | 9745 | -0.353 | -0.2568 | Yes |
| 427 | EFHC1 |  |  | 9748 | -0.355 | -0.2524 | Yes |
| 428 | SDC3 |  |  | 9756 | -0.360 | -0.2484 | Yes |
| 429 | RCN2 |  |  | 9759 | -0.364 | -0.2438 | Yes |
| 430 | NKTR |  |  | 9767 | -0.376 | -0.2397 | Yes |
| 431 | TCF4 |  |  | 9768 | -0.377 | -0.2347 | Yes |
| 432 | LRP1 |  |  | 9771 | -0.381 | -0.2299 | Yes |
| 433 | ARGLU1 |  |  | 9773 | -0.383 | -0.2250 | Yes |
| 434 | FUS |  |  | 9779 | -0.389 | -0.2204 | Yes |
| 435 | HEPN1 |  |  | 9782 | -0.395 | -0.2154 | Yes |
| 436 | ATP6AP2 |  |  | 9783 | -0.396 | -0.2102 | Yes |
| 437 | SYT11 |  |  | 9785 | -0.399 | -0.2051 | Yes |
| 438 | DDX17 |  |  | 9788 | -0.405 | -0.2000 | Yes |
| 439 | B2M |  |  | 9791 | -0.408 | -0.1948 | Yes |
| 440 | ITGB8 |  |  | 9794 | -0.418 | -0.1896 | Yes |
| 441 | CLU |  |  | 9795 | -0.418 | -0.1841 | Yes |
| 442 | LAMP2 |  |  | 9796 | -0.419 | -0.1786 | Yes |
| 443 | KANSL1 |  |  | 9797 | -0.421 | -0.1731 | Yes |
| 444 | SPARCL1 |  |  | 9798 | -0.423 | -0.1675 | Yes |
| 445 | CANX |  |  | 9804 | -0.431 | -0.1624 | Yes |
| 446 | SAT1 |  |  | 9807 | -0.445 | -0.1568 | Yes |
| 447 | VMP1 |  |  | 9817 | -0.489 | -0.1513 | Yes |
| 448 | APP |  |  | 9819 | -0.495 | -0.1449 | Yes |
| 449 | GPM6A |  |  | 9820 | -0.499 | -0.1383 | Yes |
| 450 | SLC1A3 |  |  | 9823 | -0.509 | -0.1319 | Yes |
| 451 | PTX3 |  |  | 9827 | -0.517 | -0.1254 | Yes |
| 452 | DDR1 |  |  | 9829 | -0.524 | -0.1186 | Yes |
| 453 | CD9 |  |  | 9832 | -0.544 | -0.1117 | Yes |
| 454 | WSB1 |  |  | 9836 | -0.554 | -0.1047 | Yes |
| 455 | MATN2 |  |  | 9837 | -0.571 | -0.0972 | Yes |
| 456 | ADGRG1 |  |  | 9851 | -0.654 | -0.0900 | Yes |
| 457 | PRCP |  |  | 9855 | -0.712 | -0.0810 | Yes |
| 458 | NOVA1 |  |  | 9858 | -0.741 | -0.0715 | Yes |
| 459 | MEG3 |  |  | 9859 | -0.749 | -0.0617 | Yes |
| 460 | SEMA5A |  |  | 9860 | -0.749 | -0.0519 | Yes |
| 461 | KCNQ1OT1 |  |  | 9861 | -0.751 | -0.0420 | Yes |
| 462 | NCAM1 |  |  | 9862 | -0.751 | -0.0321 | Yes |
| 463 | NEAT1 |  |  | 9866 | -0.816 | -0.0217 | Yes |
| 464 | SNTG1 |  |  | 9867 | -0.828 | -0.0109 | Yes |
| 465 | PTPRZ1 |  |  | 9869 | -0.918 | 0.0011 | Yes |
Table: GSEA details [plain text format]

  

Fig 2: DOROSZKO\_U3180MG\_CLUSTER\_2: Random ES distribution      
 Gene set null distribution of ES for **DOROSZKO\_U3180MG\_CLUSTER\_2**
